# NK cell line modified to express a potent, DR5 specific variant of TRAIL, show enhanced cytotoxicity in ovarian cancer models

**DOI:** 10.1016/j.heliyon.2024.e34976

**Published:** 2024-07-19

**Authors:** A.M. Sheedy, N. Burduli, A. Prakash, M. Gurney, S. Hanley, H. Prendeville, S. Sarkar, J. O'Dwyer, M. O'Dwyer, E.B. Dolan

**Affiliations:** a Biomedical Engineering, School of Engineering, College of Science and Engineering, University of Galway, Ireland; b CÚRAM, Centre for Research in Medical Devices, University of Galway, Galway, Ireland; c Apoptosis Research Centre, University of Galway, Galway, Ireland; d Center for Hematology Regenerative Medicine (HERM), Karolinska Institutet, Stockholm, Sweden; e Flow Cytometry Core Facility, University of Galway, Galway, Ireland; f ONK Therapeutics Inc, Galway, Ireland

## Abstract

**Objective:**

Ovarian cancer is a lethal gynaecological malignancy with unsatisfactory 5 year survival rates of 30–50 %. Cell immunotherapy is a promising new cancer treatment where immune cells, such as Natural Killer (NK) cells, are administered to enable the patient to fight cancer through direct cytotoxicity. NK cells orchestrate an adaptive immune response by enabling the release of tumour antigens. NK cell cytotoxicity and effector responses are largely driven by TRAIL engagement. In this study we investigated the cytotoxic potential of a human NK cell line that were modified to express a potent DR5 specific TRAIL variant. We hypothesised that this modification would enhance NK cell cytotoxicity against TRAIL sensitive and resistant ovarian cancer cell lines *in vitro*.

**Methods:**

KHYG-1 human NK cells were modified with a TRAIL variant targeting DR5 (TRAILv-KHYG-1). Human ovarian cancer cell lines, OVCAR-3 and SKOV-3, were cultured with modified or non-modified NK cells at different effector:target (E:T) ratios for 4 or 16 h. Apoptosis was assessed by Annexin-APC and 7-AAD and measured using flow cytometry. Apoptotic cells were defined as annexin V 7-AAD double positive. Cytokine expression was measured by multiplex ELISA, and analysed by flow cytometry.

**Results:**

Modified and non-modified NK cells significantly reduced OVCAR-3 cell viability as compared to OVCAR-3 cells that were cultured alone after 4 and 16 h treatment. OVCAR-3 cell viability was reduced after treatment with 1:1 E:T ratio with TRAILv-KHYG-1 cells after 16 h. On the contrary, neither NK cell line had any effect of SKOV-3 cell viability despite SKOV-3 cells having more DR5 surface expression compared to OVCAR-3 cells.

**Conclusions:**

TRAILv-KHYG-1 cells significantly reduced OVCAR-3 cell viability as compared to non-modified NK cells. However, no significant reduction in viability was observed when SKOV-3 cell were cultured with either NK cells, despite having more DR5 surface expression compared to OVCAR-3 cells. These data indicate that mechanisms other than DR5 expression drive TRAIL resistance in ovarian cancer.

## 1 Introduction

Ovarian cancer is the most lethal gynaecological cancer with a global 5 year survival rate of 30–50 % [1]. In 2022 an estimated 32,000 women died from ovarian cancer in EU countries, with an incidence rate of 4.32/100,000 [2]. The three main reasons for the high mortality are late detection, resistance to platinum-based chemotherapies and a limited therapeutic dose reaching the target site [3,4]. Gold standard treatment includes cytoreductive surgery in conjunction with a platinum based chemotherapy regime [3]. However, 80 % of women will relapse within 18 months [5] and eventually develop platinum-resistant disease [3]. Second-line treatment is administered after the initial treatment has failed, stopped working, or has side effects that are no longer tolerated. The response rate to current non-platinum agents is <20 % [[6], [7], [8]]. Women with relapsed ovarian cancer have a median survival of just 32 months [9]. Therefore, there is an urgent need for innovative and effective therapeutic strategies.

Cellular immunotherapy is a new approach where immune cells are administered to fight cancer [4]. Natural killer (NK) cells are innate lymphoid cells that possess both cytotoxicity and cytokine producing effector functions which enables highly specific killing of virally infected or transformed cells [10]. As such they play an integral role in mediating anti-cancer activity [11]. NK cells are a promising cell immunotherapy, as their activation occurs independent of prior antigen engagement or pre-sensitisation [12] to targets. Importantly, NK cells can serve as readily available off-the-shelf immunotherapy owing to their allogeneic nature. They do not require a human leukocyte antigen match and pose no risk of graft-vs-host disease [13].

NK cell activation is tightly regulated by engagement of activation and inhibitory receptors on the NK cell surface [14]. Healthy cells constitutively express major histone class (MHC) class 1 molecules, which bind inhibitory receptors on the NK cell surface, tolerising the NK cells towards healthy host tissue and preventing damage. Since T cell recognition is dependent on MHC [15], cancer cells frequently downregulate MHC molecules to escape T cell killing. Consequently, cancer cells render themselves susceptible to killing by NK cells, which are no longer inhibited via their inhibitory Killer-cell Immunoglobulin-like Receptors (KIRs). Recognition of stress induced ligands (e.g. MHC class I chain-related protein (MIC)A/B, UL16-binding proteins) by activating receptors on NK cells can also lead to activation of NK cells [12]. Stress ligands such as MHC I chain-related proteins A and B [16,17], UL16-binging proteins [16,18], and Tumour Necrosis Factor (TNF) - related apoptosis-inducing ligand (TRAIL) – R1/R2 ligands [[18], [19], [20]] are expressed at higher levels by cancer cells compared to normal cells.

Once an NK cell commits to killing a target cell, it directly releases perforin and granzyme into the tumour-immune synapse, inducing intrinsic apoptosis and cell lysis. Initially NK cell killing occures by granzyme B-dependent killing [21], whereas later cytolytic events are death receptor pathway-mediated, via Fas and TRAIL expressed by NK cells [21]. NK cells can release cytokines, such as Interferon Gamma (IFN-γ) and TNF, important for promoting a subsequent adaptive immune response as well as recruiting macrophages, dentritic cells and native NK cells to the target site [14], [22]. Finally, engagement of CD16 leads to strong activation, which can over-ride inhibitory inputs leading to antibody dependent cellular cytotoxicity (ADCC). NK cell killing via the death receptor pathway involves the binding of Fas Ligand (FasL) or TRAIL to their cognate receptors on tumour surfaces, Fas and Death Receptor (DR)4/DR5, respectively.

NK cell function in ascites and peripheral blood of ovarian cancer patients was impaired when compared to healthy controls [23]. This is due to immunosuppressive cytokines (TGF-B, IL-6, -8, -10), chronic activation and exhaustion, shredding of inhibitory ligands, downregulation of CD16 and/or regulatory cells or other immune modulators [[24], [25], [26], [27], [28]]. In 1982, treatment with human leukocyte interferon in patients with advanced disease resulted in enhanced NK cell activity, with some patients experiencing no disease progression for up to one year [29]. These findings provided a foundational basis for the development of NK cell therapies. A pivotal study by Geller et al., 2013 [30] demonstrated that the intraperitoneal (IP) administration of human NK cells in combination with the cytokine IL-15 effectively eradicated intraperitoneal ovarian cancer in xenograft mouse models. Following from this a number of preclinical *in vivo* studies have found that (NOD-scid IL2rgnull) NSG mouse models of ovarian cancer was responsive to treatment with IP NK cells [[31], [32], [33]]. However, identifying optimal tumour antigen target(s) which allows reliable discrimination between healthy and cancerous cells is challenging. Therefore, the identification of alternate, non-antigen targets that are widely expressed on ovarian cancer cells would be desirable.

We have identified a receptor in the TRAIL family as a promising target for NK cell based immunotherapy. Cancer cells express five membrane bound TRAIL receptors: three decoy receptors (DcR; Membrane bound DcR1, DcR2 and soluble OPG) and two active death receptors (DR; DR4 and DR5) [34]. Binding of TRAIL to decoy receptors on cancer cells impedes apoptotic signals, while its binding to active receptors induces caspase-mediated apoptosis, leading to programmed cell death [35], with no off-target cytotoxicity. Additionally, TRAIL can be cleaved from the membrane to produce soluble trail (sTRAIL). Although TRAIL is a very promising target to induce apoptosis in tumour cells, there are limitations. Cellular resistance to TRAIL has been linked to inhibitors of the apoptosis proteins, such as cellular FLICE-like inhibitor protein (c-FLIP) [35,36] and inhibitors of apoptosis protein (IAPs) [36]. However, it is not known if targeted binding of TRAIL resistant cells by NK cells could aid in overcoming said cellular resistance.

Although NK cells naturally express TRAIL [37], modifying NK cells with a novel, potent TRAIL variant specifically targeting the most abundant DR on the ovarian cancer cell surface could increase potency and specificity. Not only could this increase specific binding to the DR but also increase the binding affinity compared to wild type (WT) TRAIL. Arts et al. found DR5 to be expressed on the surface of 74 % of tumours after treatment with platinum based chemotherapies [19], however the surface expression of DRs does not guarantee apoptosis [35]. A recent study investigating secretory TRAIL-armed NK cell therapy against colorectal cancer [38], showed that these NK cells could infiltrate and induce apoptosis via the TRAIL pathway in mouse peritoneal tumours as well as inhibit tumour growth. There are a number of clinical trials investigating monoclonal antibodies targeted towards DR4 or DR5 (NCT00521404, NCT02991196, NCT03576131) and Chimeric Antigen Receptor (CAR) T cells modified to express either DR4 or DR5 (NCT03941626, NCT00923390) highlighting its promise as a clinical target.

**Fig. 1:**
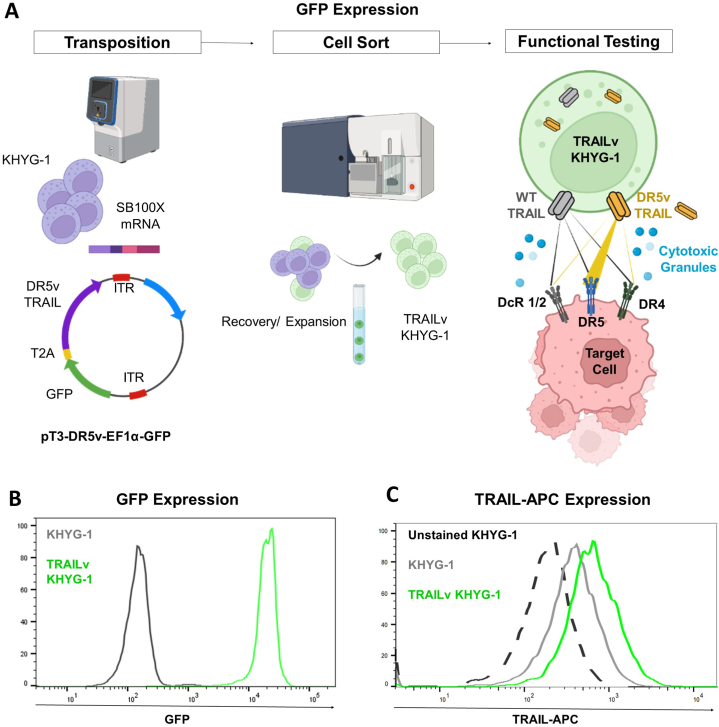
Generation of TRAILv-KHYG-1 cells. **A.** Schematic depicting the generation of DR5v KHYG-1 cells using the sleeping beauty transposon system and cell sorting for GFP expressing cells. **B.** Flow cytometric confirmation of GFP positivity in DR5v KHYG-1 cells compared to parental KHYG-1 cells post cell sorting. **C.** Fluorescent microscopy analysis of intracellular RIK2-APC staining depicting increased signal for TRAIL relative to parental and unstained KHYG-1 cells. ITR = inverted terminal repeat, WT = wild-type, DR4 = TRAILR1, DR5 = TRAILR2, DcR1 = Decoy receptor 1, DcR2 = Decoy receptor 2.

In this study, we explored the cytotoxicity of a human NK cell line genetically modified to improve DR5 targeting (TRAILv-KHYG-1 cells) against human ovarian cancer cell lines, OVCAR-3 and SKOV-3 (Fig. 1A) *in vitro*.

## 2 Material and methods

### 2.1 Modification of KHYG-1 cells to express TRAIL with high affinity for DR5

For KHYG-1 transposition, gene block fragments (Integrated DNA technologies™) were synthesized for a TRAIL variant (E195R/D269H) with enhanced DR5 receptor specificity and binding affinity, as previously reported [39]. This sequence was cloned into the sleeping beauty plasmid pT3-Neo-EF1α-GFP, gifted from Martin Bonamino (Addgene plasmid # 69134; http://n2t.net/addgene:69134; RRID:Addgene_69134), to generate pT3-EF1α-GFP-DR5vTRAIL (Fig. 1). The Sleeping Beauty (SB) transposon system is a natural gene delivery vehicle that can stably integrate a gene of interest into the genome, thereby providing a long-term or possibly permanent therapeutic [40]. One advantage of transposon systems over viral vectors is having no upper limit for cargo size for gene(s) of interest. Yet, the transfection efficiency of transposon vectors is still size-sensitive so the smaller the gene of interest to be integrated, the higher the transposition success. The immunogenicity of transposon vectors is similar to plasmid-based expression vectors and less than viral vectors by nature. Even though the production time in good manufacturing process is currently comparable with viral vectors, the GMP production cost of non-viral vectors is significantly cheaper. While there have been some recent concerns raised about the safety of transposon systems, Sleeping Beauty has an almost completely random integration pattern, which may offer increased safety in patients over other transposon systems [[40], [41], [42], [43]]. The relatively short persistence of NK cells should decrease further the risk of secondary malignancy. KHYG-1 cells (identity confirmed by STR sequencing (Eurofins Genomics™)), were harvested for electroporation in log-phase growth. 2.5 × 10^6^ KHYG-1 cells were electroporated with a cargo containing pT3-EF1α-GFP-T2A-DR5vTRAIL and SB100X transposase mRNA, using the Maxcyte™ GT flow transfection system. Upon recovery to log-phase growth and after 14-days, a distinct population expressing GFP (representing the transposed cells) was isolated by fluorescence-activated cell sorting using a BD FACS Aria™ II cell sorter. Intracellular staining for TRAIL was conducted by incubation of KHYG-1 first fixing cells in 2 % PFA for 10 min. This was followed by permeabilization in 0.1 % saponin for 10 min, prior to incubation with TRAIL Ab RIK2. APC, Biolegend 308209) in 0.1 % saponin.

### 2.2 NK cell cytotoxicity against ovarian cancer cells

Tag-It Violet (Biolegend® 425101) labelled OVCAR-3 (JCRB1609) or SKOV-3 (JCRB1549) ovarian cancer cells (targets) were cultured with non-modified (KHYG-1) or modified (TRAILv-KHYG-1) NK cells (effectors) at 0.1:1, 1:1, 5:1 or 10:1 effector-to-target (E:T) ratios. After 4 or 16 h, samples were stained and analysed by flow cytometry on a Cytek Bioscience Northern Lights 3000 spectral flow cytometer, using APC Annexin V Apoptosis Detection Kit with 7-AAD (Biolegend® 640930). A Multiplex ELISA (Custom Human GeniePlex MultiPlex Assay detecting soluble TRAIL and cytokines, IFNγ, granzyme B, and granzyme A) was performed using the supernatant from OVCAR-3 and SKOV-3 cells after 16 h co-culture with KHYG-1 or TRAILv-KHYG-1 NK cells. Cytokine expression was measured by flow cytometry using a BD FACS Canto II™ flow cytometer.

### 2.3 Membrane bound DR expression

OVCAR-3, SKOV-3, KHYG-1 and TRAILv-KHYG-1 cells were fluorescently tagged with Propidium iodide (PI) labelled DR4, DR5 and DcR1. Mean Fluorescent Intensity (MFI) was measured using flow cytometry on a Cytek Bioscience Northern Lights 3000 spectral flow cytometer.

### 2.4 Statistical analysis

Flow cytometry data was analysed using FlowJo v10 (Treestar Inc.). Statistical analysis (one way anova and unpaired *t*-test) was carried out using Prism Version 8 (n = 3–6/group).

## 3 Results

### 3.1 Development of NK cell immunotherapy targeted to DR5

Flow cytometry was used to confirm that TRAILv-KHYG-1 NK cells express GFP (Fig. 1B) and detect an increase in the level of TRAIL expression in intracellularly stained TRAILv-KHYG-1 NK cells relative to KHYG-1 NK cells. Upon intracellular staining as measured by immunofluorescence (representative histograms depicted in Fig. 1B and C), TRAILvKHYG-1 cells express GFP and have higher expression of TRAIL as compared to unmodified KHYG-1 cells. No obvious fratricide was observed.

### 3.2 KHYG-1 and TRAILv-KHYG-1 cells display enhanced cytotoxicity against OVCAR-3, but not SKOV-3 cells

**Fig. 2:**
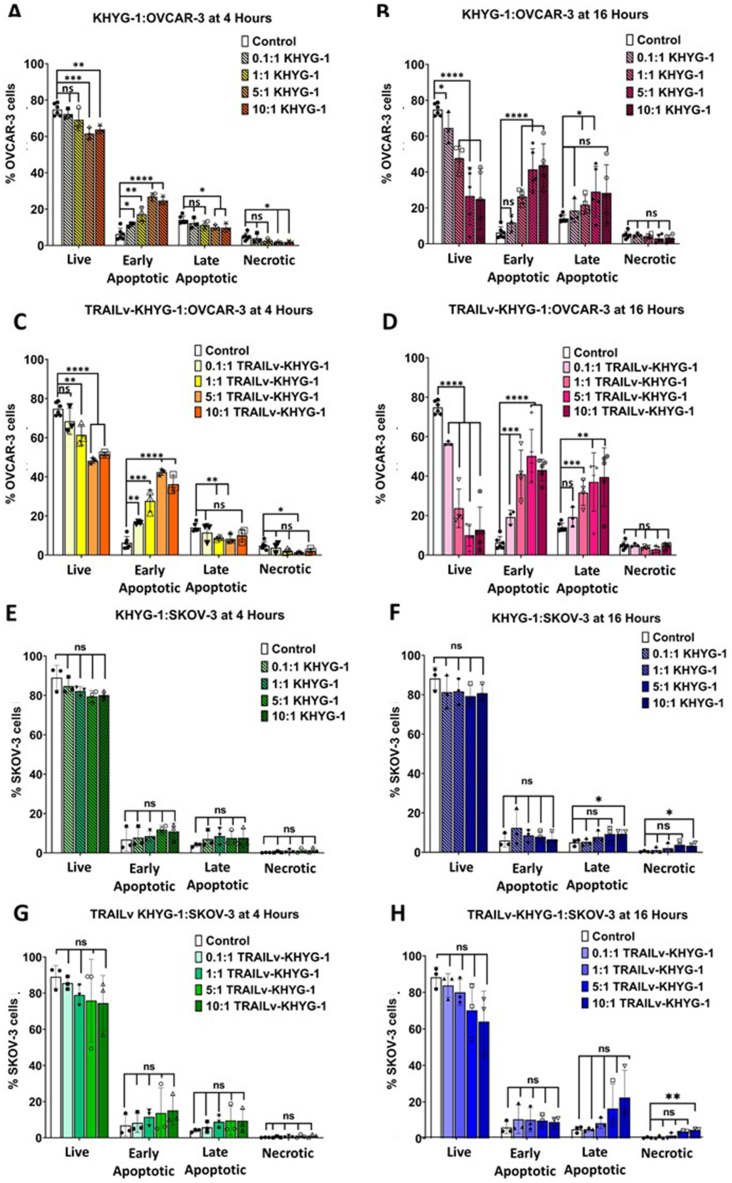
Apoptosis and necrosis measured by Annexin V APC/7AAD staining of OVCAR-3 (A–D) or SKOV-3 (E–H) cells co-cultured with KHYG-1 and TRAILv-KHTG-1 NK cells under various conditions. Quadrant gates define live cells (7AAD^−^ Annexin V APC^−^), early apoptotic cells (7AAD^−^ Annexin V APC^+^), late stage apoptotic cells (7AAD^+^ Annexin VAPC^+^) and necrotic cells (7AAD^+^ Annexin V APC^−^). **A:** Percentages of OVCAR-3 cells in each quadrant co-cultured with 0.1:1, 1:1, 5:1 and 10:1 ET ratios of KHYG-1 cells for 4 h, **B:** OVCAR-3 cells co-cultured with KHYG-1 cells at the same E:T ratios for 16 h, **C & D:** SKOV-3 cells co-cultured with TRAILv-KHYG-1 cells at the same E:T ratios for 4 and 16 h respectively. **E:** Percentages of SKOV-3 cells in each quadrant co-cultured with 0.1:1, 1:1, 5:1 and 10:1 ET ratios of KHYG-1 cells for 4 h, **F:** SKOV-3 cells co-cultured with KHYG-1 cells at the same E:T ratios for 16 h, **G & H:** SKOV-3 cells co-cultured with TRAILv-KHYG-1 cells at the same E:T ratios for 4 and 16 h respectively. Data shown as mean ± SD, n = 3–6. Statistics by unpaired *t*-test, ns = not significant, *p < 0.05, **p < 0.005, ***p < 0.001, ****p < 0.0001.

KHYG-1 cells significantly reduced OVCAR-3 cell viability at 5:1 and 10:1 E:T ratios after 4 h, and increased early apoptosis at all E:T ratios (Fig. 2A, ST1). This effect intensified at 16 h, showing significant reductions in viability and increases in early and late apoptosis at all E:T ratios (Fig. 2B, ST1). No significant differences in necrosis were observed at either time points. Similarly, TRAILv-KHYG-1 cells significantly reduced OVCAR-3 cell viability at 1:1, 5:1, and 10:1 E:T ratios after 4 h, and increased early apoptosis at all ratios (Fig. 2C, ST1). Similarly, this effect intensified at 16 h, with further reductions in viability and increases in early and late apoptosis at all E:T ratios (Fig. 2D, ST1). No significant differences in necrosis were observed at either 4 or 16 h compared to controls.

A minor E:T ratio response was evident when SKOV-3 cells were co-cultured with KHYG-1 cells at 4 h (Figs. 2E) and 16 h (Fig. 2F). No significant differences in the proportion of live cells, early apoptosis, late apoptosis, or necrosis were found at 4 h (Fig. 2E). After 16 h, a significant increase in late apoptosis and necrosis was observed at the 10:1 ratio, with no significant reduction in viability or increase in early apoptosis (Fig. 2F). In SKOV-3 cells co-cultured with TRAILv-KHYG-1 cells, a more noticeable but non-significant E:T ratio response was seen (Fig. 2G and H). At 4 h, no significant reduction in the proportion of live cells or increase in apoptosis or necrosis was observed (Fig. 2G). At 16 h, no significant reduction in viability or increase in apoptosis was observed, but a significant increase in necrosis was noted at the 10:1 ratio (Fig. 2H, ST1).

These data indicate that both non-modificed KHYG-1 and modified TRAILv KHYG-1 cell lines can reduce OVCAR-3, but not SKOV-3 cell viability *in vitro*. Representative flow cytometry density plots in Supplementary Fig. 1.

### 3.3 Direct comparative analysis of cytotoxic effects of non-modified and TRAIL-modified KHYG-1 NK cells on OVCAR-3 and SKOV-3 ovarian cancer cell lines

**Fig. 3:**
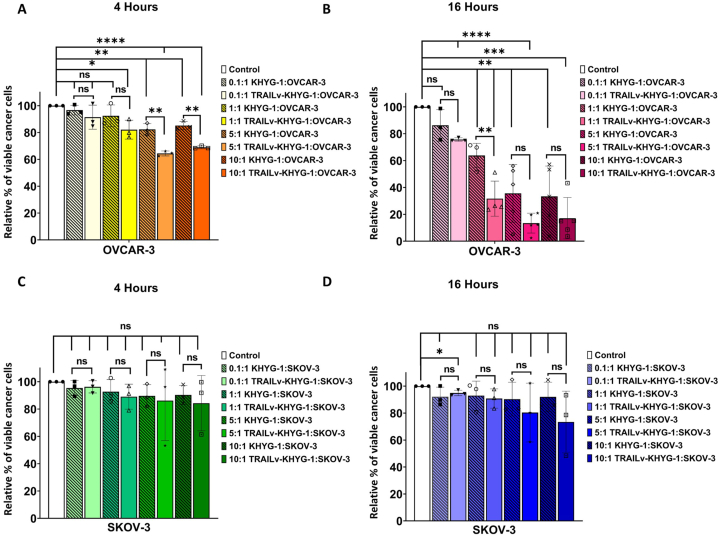
OVCAR-3 and SKOV-3 cells were normalised against their control to determine the relative percentage of live cells are various E:T ratios. The relative percentage of live OVCAR-3 cells treatment with 0.1:1, 1:1, 5:1 and 10:1 ET ratios of KHYG-1 or TRAILv-KHYG-1 cells for **A:** 4 h or **B:** 16 h. The relative percentage of live SKOV-3 cells treatment with 0.1:1, 1:1, 5:1 and 10:1 ET ratios of KHYG-1 or TRAILv-KHYG-1 cells for **C:** 4 h or **D:** 16 h. Data shown as mean ± SD, n = 3–6. Statistics by unpaired *t*-test, ns = not significant, *p < 0.05, **p < 0.005, ***p < 0.001, ****p < 0.0001.

Next, we directly compared the ability of the NK cells to reduce OVCAR-3 and SKOV-3 viability by quantifying the live (7AAD^−^ Annexin V APC^−^) cells and normalising against the cancer cells alone to allow for direct comparison between OVCAR-3 and SKOV-3 cells (Fig. 3). We directly compared KHYG-1 to TRAILv-KHYG-1 cells to determine if the TRAIL modification enhances the cytotoxic efficacy of NK cells against ovarian cancer cell lines. At 4 h, when directly comparing the non-modified KHYG-1 cells to their modified counterparts at each E:T ratio, the % viability was significantly reduced for 5:1 (p < 0.005) and 10:1 (p < 0.005) cultured with TRAILv-KHYG-1s, indicating that the modified cells were more potent (Fig. 3A). At 16 h, when directly comparing the KHYG-1s to TRAILv-KHYG-1s at each E:T ratio, the % viability was significantly reduced for 1:1 (p < 0.005) after treatment with TRAILv-KHYG-1 (Fig. 3B).

In contrast, an E:T ratio response was not clearly evident in SKOV-3 cells at either time point (Fig. 3C and D). At 4 and 16 h there was no significant reduction in % viability compared to control or when directly comparing the KHYG-1 cells to TRAILv-KHYG-1 cells at each E:T ratio (Fig. 3C and D).

These comparisons revealed that TRAILv-KHYG-1 cells significantly reduced the viability of OVCAR-3 cells at multiple E:T ratios, whereas no significant differences were observed in SKOV-3 cells, indicating that the modified NK cells were more effective against OVCAR-3 cells. Therefore these data indicate that OVCAR-3 cells are more sensitive to killing by both KHYG-1 and TRAILvKHYG-1 cells.

### 3.4 Differential secretion of granzymes, INFgamma, and sTRAIL by non-modified and TRAIL-modified KHYG-1 NK cells in response to OVCAR-3 and SKOV-3 ovarian cancer cell lines

Given the differences in cell viability between OVCAR-3 and SKOV-3 cell lines, we next analysed cytokine expression in the supernatants of these groups to determine if the modification of KHYG-1 cells resulted in differential cytokine production.

**Fig. 4:**
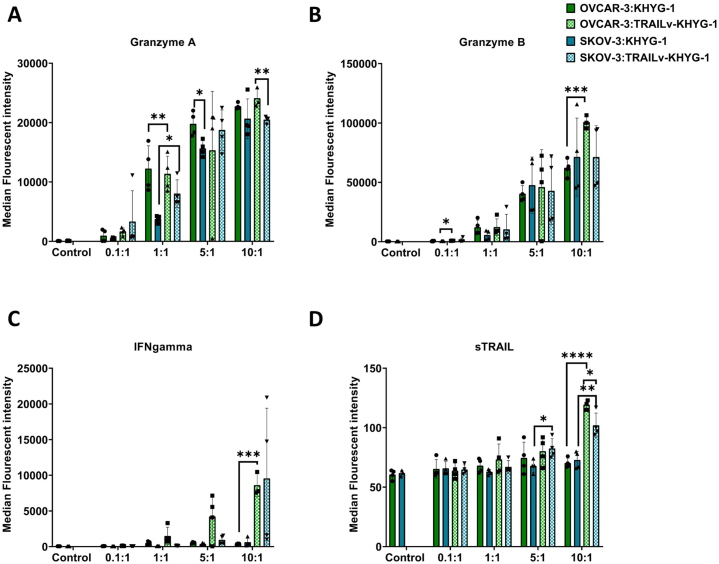
The median fluorescent intensity of **A:** Granzyme A, **B:** Granzyme B, **C:** INFgamma**,** and **D:** sTRAIL within supernatant of OVCAR-3 cells or SKOV-3 co-cultured with 0.1:1, 1:1, 5:1 and 10:1 ET ratios of KHYG-1 or TRAILv-KHYG-1 cells for 16 h. Data shown as mean ± SD, n = 2, in duplicate. Statistics by unpaired *t*-test, ns = not significant, *p < 0.05, **p < 0.005, ***p < 0.001, ****p < 0.0001.

We examined the secretion of granzymes, INF-γ, and sTRAIL which play critical roles in NK cell-mediated cytotoxicity and immune regulation against cancer cell lines *in vitro* and *in vivo*^14^*.* Granzymes (A and B) are serine proteases that induce apoptosis in target cells, INF-γ is a cytokine that activates immune responses and enhances antigen presentation, and sTRAIL induces apoptosis in cancer cells by binding to death receptors. The supernatants of OVCAR-3 and SKOV-3 cells after 16 h of culture with either KHYG-1 or TRAILv-KHYG-1 cells were analysed using a multiplex ELISA. Granzyme A secretion increased with increasing E:T ratios across all groups (Fig. 4A). TRAILv-KHYG-1 cells secreted more Granzyme A than KHYG-1 cells, significantly at 1:1 for both OVCAR-3 (p < 0.005) and SKOV-3 (p < 0.05) cells (Fig. 4A). Both NK cells tended to secrete more Granzyme A when cultured with OVCAR-3 cells, significantly so at 5:1 KHYG-1 (p < 0.05) and 10:1 TRAILv-KHYG-1 (p < 0.005).

Similarly, secretion of granzyme B (Fig. 4B) increased with increasing E:T ratios. However, Granzyme B was secreted at a similar level by both NK cells for all conditions except 10:1 OVCAR-3 cultured with TRAILv-KHYG-1 cells (p < 0.001). Neither NK cells secreted significantly more Granzyme B when cultured with OVCAR-3 cells compared with SKOV-3 cells.

Secretion of INF-γ (Fig. 4C) by TRAILv-KHYG-1 cells against OVCAR-3 cells increased with increasing E:T ratios. Significantly more INFγ was secreted against OVCAR-3 at 10:1 by TRAILv-KYG-1 compared with KHYG-1 cells (p < 0.001). Interestingly, the only E:T ratio resulting in an increase in INF-γ against SKOV-3 cells was 10:1 with TRAILv-KHYG-1 cells.

Finally secretion of sTRAIL (Fig. 4D) by KHYG-1 cells remained consistent among all E:T ratios for both OVCAR-3 and SKOV-3 cells. At 10:1, an increase in sTRAIL was observed for both OVCAR-3 and SKOV-3 cells cultured with TRAILv-KHYG-1 cells. This was significantly more than KHYG-1 cells for OVCAR-3 (p < 0.0001) and SKOV-3 (p < 0.05) cells.

As we found little significant difference in cytokine expression between the non-modified and modified cells, we examined the surface expression of DR4, DR5, and DcR1 to determine if these markers contribute to the differential cytotoxicity observed.

### 3.5 Analysis of DR4, DR5, and DcR1 surface expression in OVCAR-3 and SKOV-3 cells

**Fig. 5:**
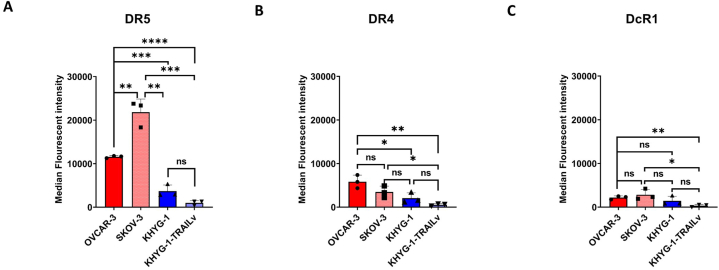
The Median fluorescent intensity of OVCAR-3, SKOV-3, KHYG-1 and TRAILv-KHYG-1 cells tagged with **A:** DR5-PI, **B:** DR4-PI, **C:** DcR1-PI. Data shown as mean ± SD, n = 3. Statistics by unpaired *t*-test, ns = not significant, *p < 0.05, **p < 0.005, ***p < 0.001, ****p < 0.0001.

Due to the significant differences in cytotoxicity when OVCAR-3 and SKOV-3 cells were cultured with NK cells, we aimed to identify whether there was any correlation between cytotoxicity and surface expression of DR4, DR5 or DcR1, cell surface markers typically found on ovarian cancer cells (Fig. 5A–C). DR5, DR4 or DcR1 expression on the surface of OVCAR-3, SKOV-3, KHYG-1 and TRAILv-KHYG-1 cells was measured with flow cytometry. OVCAR-3 cells had significantly lower surface expression of DR5 (Fig. 5A) compared with SKOV-3 cells (p < 0.005). There was no significant difference in DR4 surface expression of OVCAR-3 compared with SKOV-3 (Fig. 5B). Finally when examining the Decoy receptor expression (DcR1), there was no significant difference was observed between OVCAR-3 and SKOV-3 cells. When examining the expression of DR5 (Fig. 5A), DR4 (Fig. 5B) and DcR1 (Fig. 5C) on both KHYG-1 and TRAILv-KHYG-1 cells, negligible expression was measured. These findings suggest that the differential cytotoxicity observed is not directly related to the surface expression levels of DR4, DR5, or DcR1 on the ovarian cancer cells.

## 4 Discussion

There is an urgent need for innovative and effective therapeutic targets and strategies for the treatment of ovarian cancer, as the current gold standard treatment provides 5 year survival rates <50 % [1] after first line treatment, and as low as 10–15 % after second line treatment of platinum resistant disease [4]. This study builds on the initial success of unmodified NK cells against ovarian cancer *in vivo* [30,31,44] and highlights the utility in modifying NK cells to specifically target receptors expressed on ovarian cancer cells surface such as TRAIL. TRAIL is commonly expressed on NK cells. It can engage with active or decoy death receptors (DR4, DR5, or DcR1) on target cells. Following engagement with DR5 on a target cell, it induces apoptosis *in vitro* and *in vivo* [34,45] with little to no off-target cytotoxicity observed. Despite high expression of DR5 on ovarian cancer patient samples [19,46], resistance to TRAIL induced apoptosis is well reported [35]. Resistance may be due to overexpression of decoy receptors, over expression of c-FLIP [35,36] or IAPs [36]. However, it's unknown whether TRAIL resistance can be overcome by specific targeting of DR5 and whether cytolytic granule released can significantly contribute to apoptosis in the event of persistent TRAIL resistance. We hypothesised that by selectively targeting DR5 on ovarian cancer cell surfaces, using a potent and specific TRAIL variant with higher binding affinity than WT TRAIL [39,47], we can avoid competition with other death receptors, especially decoy receptors, and exert a greater cytotoxic. Here, we developed a novel human NK cell immunotherapy targeted to DR5 (TRAILv-KHYG-1) and tested its cytotoxicity against human ovarian cancer cell lines, OVCAR-3 and SKOV-3.

Although we have not directly looked at safety in this study, a previous study by Stikvoort et al., 2021 [48] looked at the safety and efficacy of a modified KHYG-1 cell, a CD38-specific Chimeric Antigen Receptor Expressing KHYG-1 cell via retroviral transduction, in the context of multiple myeloma (MM). They found that their modified KHYG-1 NK cells effectively mediate anti-MM activity, while there is low or no cytotoxicity against nonmalignant cells especially against T, and NK cells, with one remarkable exception: the elimination of CD38^+^ regulatory T cells. Another safety advantage of NK cells over other immune cells, such as T cells, is that NK cells are short lived, meaning that any adverse effects associated with NK cells, would be significantly shorter than those of T cells. Additionally, other NK cell lines such as NK-92, have been irradiated prior to being used in clinical trials and have been found safe. This option could be expored with KHYG-1 cells.

OVCAR-3 [49] and SKOV-3 [50] are both derived from serous ovarian adenocarcinoma, which represent the most common and aggressive subtype [[51], [52], [53], [54]]. They are both extensively characterised and tested *in vitro* [49,[55], [56], [57], [58], [59], [60], [61]] and *in vivo* [58,[61], [62], [63], [64], [65]] for many years. Through reviewing literature of recombinantTRAIL (rTRAIL) therapies used to treat ovarian cancer, we identified that OVCAR-3 and SKOV-3 are susceptible and resistant to rTRAIL killing respectively [66,67]. For these reasons OVCAR-3 and SKOV-3 were chosen as appropriate cell lines to test our therapy. We found that KHYG-1 and TRAILv-KHYG-1 produced a significant cytotoxic effect on OVCAR-3 cells after 4 and 16 h exposure (Fig. 2). A dose-response was observed with decreasing % of live OVCAR-3 cells with increasing E:T ratios. A significant difference was observed between the KHYG-1 cells and TRAILv-KHYG-1 cells at 5:1 and 10:1 at 4 h and 1:1 at 16 h, where the modified cells produced a greater cytotoxic effect indicating a more potent therapy. A promising result from this study was that the percentage reduction in viability of OVCAR-3 cells was 51.08 ± 8.47 %, after treatment with 1:1 E:T ratio with TRAILv-KHYG-1 cells. The observed efficacy of our TRAILv-KHYG-1 cell therapy is noteworthy when compared to other published therapies. *In vitro* studies have reported viability reductions ranging from 10 % to 40 % for the same (1:1) or higher (2.2:1) E:T ratios with modified NK cells [30,31,33]. By achieving a viability reduction of over 50 % at a 1:1 E:T ratio, our therapy demonstrates competitive or superior effectiveness compared to these benchmarks. We also observed an increasing trend in granzyme A and B secretion with increasing E:T ratio for OVCAR-3 co-cultured with both NK cell therapies (Fig. 4). A dose response in INF-γ secretion was observed when OVCAR-3 cells were co-cultured with TRAILv-KHYG-1 cells but not KHYG-1 cells. This trend was in agreement with Cao et al., 2020 [68] when a CAR-NK cells co-cultured OVCAR-3 and SKOV-3 cells at 1:1 E:T ratio *in vitro*. NK cells exert their cytotoxic activity through two distinct pathways. Firstly, they can release cytotoxic granules containing molecules such as perforin and granzymes. Alternatively, they can induce death receptor-mediated apoptosis by expressing molecules like TRAIL and/or Fas ligand, which engage receptors such as TRAIL-R1/-R2 or CD95/Fas on the surface of diseased cells [69]. Granzyme A functions by targeting the cell nucleus, inducing DNA damage. Importantly, apoptosis triggered by Granzyme A operates independently of the caspase cascade. On the other hand, Granzyme B contributes to apoptosis by activating caspases, such as effector caspase 3, or alternatively, it can initiate apoptosis in a caspase-independent manner by engaging Bcl-2 family proteins [70]. The elevated levels of granzymes A and B indicate the activation of cytotoxic pathways in TRAILv-KHYG-1 cells upon interaction with OVCAR-3 cells and is in agreement with Cao et al., 2020 [68]. This is a promising result as in clinical settings elevated granzyme A and B may be associated with improved outcomes in advanced, treated ovarian cancer and have been linked with survival in ovarian cancer [71,72]. An increase in production of INFγ indicates NK cell activation and plays an important role in recruiting other immune cells to the tumour site such as CD8 and Th1 cells [73]. We have shown that when TRAILv-KHYG-1 cells contact OVCAR-3 cells they increase INF-γ release in a dose dependant manor which is in agreement with Y. Lu. et al., 2013 [74]. This suggests that these cells can exert cytotoxic killing of ovarian cancer cell lines, and also may recruit other cytotoxic cells to further impede tumour growth and dissemination. This can ultimately drive a more robust immune response as described in detail in a review by Schoenborn et al., 2007 [73]. Finally, sTRAIL increases at 10:1 for TRAILvKHYG-1 cells against OVCAR-3 and SKOV-3. sTRAIL is produced by either proteolytic cleavage of membrane bound TRAIL on NK cells or cellular secretion [75]. This could lead to a greater availability of TRAIL to engage with receptors in a clinical setting.

Our results suggests that OVCAR-3 cells were killed by the multimodal effect of NK where the TRAIL pathway was engaged resulting in apoptosis. KHYG-1 cell exerted a significantly lower cytotoxic effect compared with TRAILv-KHYG-1 against OVCAR-3. The increased killing associated with TRAILv-KHYG-1 cells may be due to the higher potency of the mutated DR5v TRAIL compared with WT TRAIL, leading to a stronger pro-apoptotic signal. These data highlight the importance of designing specifically targeted treatments for targets, such as DR5, known to be highly expressed on the cancer cell surface. Our results show the potential of targeting DR5 expressing tumour cells, such as OVCAR-3 with NK cells engineered to express a potent and specific DR5 TRAIL variant, which maximizes both Granzyme mediated killing as well as apoptosis via the death receptor pathway.

Certain ovarian cancer cell lines, such as SKOV-3, are known to be TRAIL resistant [76]. In this study, SKOV-3 cells also proved to be NK resistant with minimal cytotoxicity when cultured with either KHYG-1 or TRAILv-KHYG-1 cells (Fig. 2). A minor effect was seen after treatment with TRAILv-KHYG-1 cells at 10:1 reducing viability from 88.2 ± 4.69 % to 63.97 ± 13.67 % after 16 h, although this was not significant. This was markedly less than was observed when OVCAR-3 cells are cultured with the same E:T ratios and time points (12.77 ± 10.28 % viability). While both TRAILv-KHYG-1 cells and recombinant DR5 alone induce cell death in OVCAR-3 cells, there are notable differences in their mechanisms and potential clinical implications. Here we show that TRAILv-KHYG-1 cells are modified NK cells engineered to express the tumour necrosis factor-related apoptosis-inducing ligand (TRAIL), which induces apoptosis in cancer cells upon binding to death receptors like DR5, a novel form of selectively inducing the DR5 pathway. On the other hand, recombinant DR5 is a soluble form of the DR5 receptor agonist administered directly to the cells. Similar viability have been found in other papers with cell death reported with 500 ng/mL recombinant DR5 alone for 48 h in *in vitro* models utilizing OVCAR-3 (20 % viability) and SKOV-3 (58 % viability) [66,67]. While both approaches target the same pathway, TRAILv-KHYG-1 cells may offer advantages such as targeted delivery, and potential synergy with other NK cell functions, such as cytokine secretion and direct cytotoxicity *in vivo* or in translation to clinic. When examining the cytolytic granules, granzyme A and B, released by the NK cells, we found an increased release with increasing E:T ratios (Fig. 4). Taken together our results suggest that specifically targeting the known abundant active DR on the cell surface does not guarantee apoptosis in TRAIL resistant cells, however, it does significantly increase cell death in TRAIL sensitive cells.

Finally, we investigated the presence of the death receptors ligand on the surface of the ovarian cancer and NK cells (Fig. 5). We observed significantly lower expression of DR5, DR4 and DcR1 ligands expressed on the NK cell surface, indicating their resistance to fratricide. We found 1.89 times more DR5 ligands present on the SKOV-3 cells surface than on OVCAR-3 cell surface and no significant difference in DR4 or DcR1, suggesting that the lack of induced apoptosis in SKOV-3 cells was not due to the lack of DR5 ligands availablity. This result indicates that ligand expression alone is not a determining factor in TRAIL induced apoptosis. Li et al., have shown that c-FLIP_L_ knockdown can make TRAIL-resistant ovarian cancer cells sensitive to TRAIL [76]. This is an important finding as many publications report an upregulation of DR5 in patients who have undergone chemotherapy, making DR5 a target for second line treatment. However, we have found that a DR5 upregulation alone is not enough to ensure cell death occurs and that means of reducing c-FLIP_L_ in conjunction with DR5 targeted NK cell therapy may be necessary to provide meaningful clinical translation for TRAIL resistant cells.

Although this work showed promising results for our TRAILv-KHYG-1 cell immunotherapy *in vitro*, there are some limitations. Firstly, 2D co-culture of isolated cell lines KHYG-1 with either OVCAR-3 or SKOV-3 cells provide a limited snapshot of what would happen *in vivo*, where cell signalling, immune cells recruitment and the tumour microenvironment will influence the effectiveness of the therapy. Examination of this therapy in an *in vivo* model would provide more insight into how the cells might perform in a clinical setting. Secondly, in this study, we examined cytotoxicity at both 4 and 16 h, although the most commonly used timepoint in published literature is 4 h [30,31,77,78]. Most publications note that NK cell killing peaks around 9–10 h [[79], [80], [81]] and there have been a limited number which have found peak killing to be at later time points such as 24 h [82]. Although we sought to enhance our understand of NK cell cytotoxicity by including 2 time points (4 and 16 h), a real time continuous measurement would be beneficial. Thirdly, although this study has shown the cytotoxic effect of our therapy *in vitro*, we have an incomplete understanding of what is happening at a molecular level. This could result in potential off-target effects, sub-optimal killing, inability to overcome resistance and may hinder translatability. However, this work still provides an important proof-of-concept that shows that modification of NK cells to specifically target DR5 can enhance NK cell cytotoxicity against ovarian cancer cells. To address the gap in knowledge of the mechanism of action and further improve the killing capability of these TRAILvKHYG-1 cells, future work should block or knock out the DR-5 receptor on OVCAR-3, and subsequently examine the cytotoxic effect of TRAILv-KHYG-1 cells. Literature shows that utilizing C-FLIP [76] or IAP [83] inhibitors may overcome TRAIL resistance in SKOV-3 cells. Future work could investigate these inhibitors in combination with our developed NK cell therapy to identify a potent regime for TRAIL resistant cells. Addressing these limitations would be important to confidently progress the TRAILv modification of NK cells into *in vivo* assessment and subsequently clinical trials, as TRAIL in an attractive target due to its significant upregulation post-chemotherapy treatment in ovarian cancer patients [19]. The use of KHYG-1 cell line, has provided a proof-of-concept study, where future work could focus on engineering primary NK cells to express TRAIL variants [84] for the potential future progression into a clinical setting.

## 5 Conclusion

To summarise, a novel TRAILv-KHYG-1 NK cell immunotherapy was developed and the cytotoxic effect of this therapy against OVCAR-3 and SKOV-3 ovarian cancer cells was compared with its non-modified counterpart (KHYG-1). While recombinant DR5 has shown efficacy in inducing cell death in ovarian cancer cell lines *in vitro*, its clinical translation may be limited by factors such as systemic toxicity, short half-life, and off-target effects [66,67,85]. In contrast, TRAILv-KHYG-1 cells offer the potential for targeted and sustained delivery of TRAIL specifically to tumour cells, potentially minimizing systemic side effects and enhancing therapeutic efficacy. We found TRAILv-KHYG-1 cells to be more effective at inducing OVCAR-3 cell death compared to non-modified cells at 4 and 16 h treatment. Our modified cells are potent towards OVCAR-3 cells. We also found that there was no significant reduction in SKOV-3 cell death when compared with control with modified nor non-modified KHYG-1 cells at either 4 or 16 h. This highlights that the specific targeting of active DR ligands alone is not enough to mitigate TRAIL resistance. Moreover, the ability of TRAILv-KHYG-1 cells to engage in other NK cell-mediated antitumour activities, such as ADCC and cytokine production, may further enhance their therapeutic potential *in vivo* and clinical translation.

## CRediT authorship contribution statement

**A.M. Sheedy:** Writing – review & editing, Writing – original draft, Visualization, Project administration, Methodology, Investigation, Funding acquisition, Formal analysis, Data curation, Conceptualization. **N. Burduli:** Writing – review & editing, Methodology, Investigation, Formal analysis, Data curation. **A. Prakash:** Writing – review & editing, Methodology, Investigation, Formal analysis. **M. Gurney:** Writing – review & editing, Methodology, Investigation, Formal analysis, Data curation. **S. Hanley:** Writing – review & editing, Methodology, Investigation, Data curation. **H. Prendeville:** Writing – review & editing. **S. Sarkar:** Writing – review & editing, Methodology, Investigation, Data curation. **J. O'Dwyer:** Writing – review & editing, Methodology, Investigation, Data curation. **M. O'Dwyer:** Writing – review & editing, Visualization, Validation, Supervision, Resources, Methodology, Investigation, Funding acquisition, Formal analysis, Data curation, Conceptualization. **E.B. Dolan:** Writing – review & editing, Writing – original draft, Visualization, Validation, Supervision, Software, Resources, Project administration, Methodology, Investigation, Funding acquisition, Formal analysis, Data curation, Conceptualization.

## Declaration of competing interest

The authors declare the following financial interests/personal relationships which may be considered as potential competing interests:Mark Gurney reports equipment, drugs, or supplies was provided by ONK Theraputics. Michael O'Dwyer reports a relationship with ONK Therapeutics that includes: board membership, consulting or advisory, and equity or stocks. Michael O'Dwyer has patent #US10034925B2 issued to Michael O'Dwyer. Michael O'Dwyer has patent #EP3454871B1 issued to Michael O'Dwyer. If there are other authors, they declare that they have no known competing financial interests or personal relationships that could have appeared to influence the work reported in this paper.
