# Supplementary material for: NK cell line modified to express a potent, DR5 specific variant of TRAIL, show enhanced cytotoxicity in ovarian cancer models
Source: Heliyon. 2024 Jul 19;10(15):e34976. doi: 10.1016/j.heliyon.2024.e34976 (PMC11336271; doi:10.1016/j.heliyon.2024.e34976)
Supplement: Multimedia component 1 [file mmc1.docx]

**Supplementary Table 1**: Statistical significance of OVCAR-3 and SKOV-3 treated with either KHYG-1 or TRAILv-KHYG-1 at 4 or 16 hours compared to non-treated control. E. Apop. = Early Apoptosis, L. Apop. = Late Apoptosis.

|  | | | **OVCAR-3 Cells** | |  | **SKOV-3 Cells** | |
| --- | --- | --- | --- | --- | --- | --- | --- |
|  |  |  | **KHYG-1** | **TRAILv-KHYG-1** |  | **KHYG-1** | **TRAILv-KHYG-1** |
|  | **E:T Ratio** | **Quadrant** | **Significance against control** | **Significance against control** |  | **Significance against control** | **Significance against control** |
| **4 Hours** | **0.1:1** | **Alive** | NS | NS |  | NS | NS |
|  |  | **E. Apop.** | <0.05 | <0.005 |  | NS | NS |
|  |  | **L. Apop.** | NS | NS |  | NS | NS |
|  |  | **Necrosis** | NS | NS |  | NS | NS |
|  | **1:1** | **Alive** | NS | <0.005 |  | NS | NS |
|  |  | **E. Apop.** | <0.005 | <0.001 |  | NS | NS |
|  |  | **L. Apop.** | NS | <0.005 |  | NS | NS |
|  |  | **Necrosis** | NS | NS |  | NS | NS |
|  | **5:1** | **Alive** | <0.001 | <0.0001 |  | NS | NS |
|  |  | **E. Apop.** | <0.0001 | <0.0001 |  | NS | NS |
|  |  | **L. Apop.** | <0.05 | <0.005 |  | NS | NS |
|  |  | **Necrosis** | <0.05 | <0.05 |  | NS | NS |
|  | **10:1** | **Alive** | <0.005 | <0.0001 |  | NS | NS |
|  |  | **E. Apop.** | <0.0001 | <0.0001 |  | NS | NS |
|  |  | **L. Apop.** | <0.05 | NS |  | NS | NS |
|  |  | **Necrosis** | <0.05 | NS |  | NS | NS |
| **16 Hours** | **0.1:1** | **Alive** | <0.05 | <0.0001 |  | NS | NS |
|  |  | **E. Apop.** | NS | <0.001 |  | NS | NS |
|  |  | **L. Apop.** | NS | NS |  | NS | NS |
|  |  | **Necrosis** | NS | NS |  | NS | NS |
|  | **1:1** | **Alive** | <0.0001 | <0.0001 |  | NS | NS |
|  |  | **E. Apop.** | <0.0001 | <0.001 |  | NS | NS |
|  |  | **L. Apop.** | <0.05 | <0.001 |  | NS | NS |
|  |  | **Necrosis** | NS | NS |  | NS | NS |
|  | **5:1** | **Alive** | <0.0001 | <0.0001 |  | NS | NS |
|  |  | **E. Apop.** | <0.0001 | <0.0001 |  | NS | NS |
|  |  | **L. Apop.** | <0.05 | <0.005 |  | NS | NS |
|  |  | **Necrosis** | NS | NS |  | NS | NS |
|  | **10:1** | **Alive** | <0.0001 | <0.0001 |  | NS | NS |
|  |  | **E. Apop.** | <0.0001 | <0.0001 |  | NS | NS |
|  |  | **L. Apop.** | NS | <0.005 |  | <0.05 | NS |
|  |  | **Necrosis** | NS | NS |  | <0.05 | <0.05 |
