# Supplementary material for: NK cell line modified to express a potent, DR5 specific variant of TRAIL, show enhanced cytotoxicity in ovarian cancer models
Source: Heliyon. 2024 Jul 19;10(15):e34976. doi: 10.1016/j.heliyon.2024.e34976 (PMC11336271; doi:10.1016/j.heliyon.2024.e34976)
Supplement: Multimedia component 2 [file mmc2.docx]

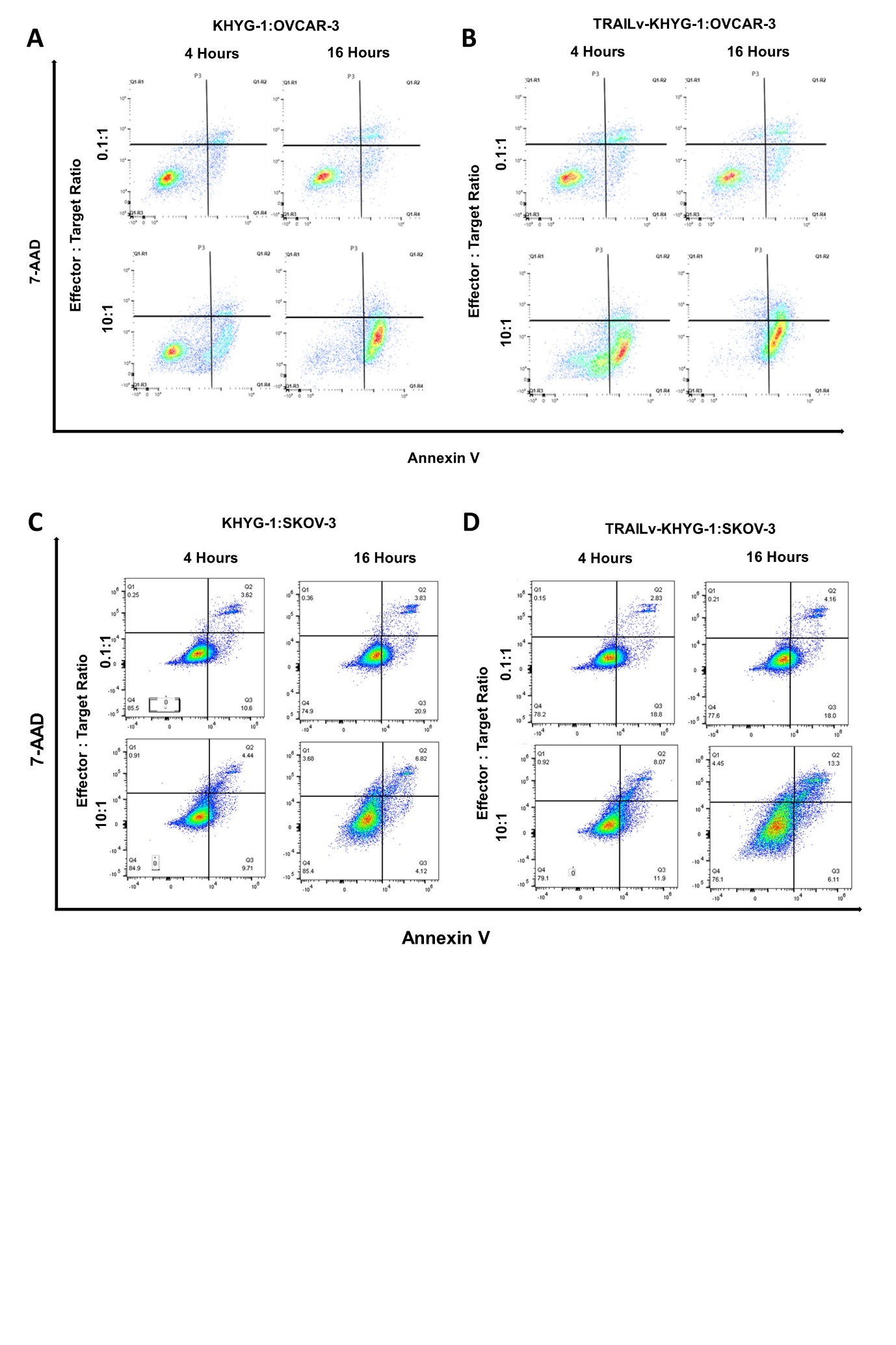
**Supplementary Figure 1 (SF1):** Representative flow cytometry pseudo colour density plots of apoptosis measured by Annexin V APC/7AAD staining of OVCAR-3 cells (**A, B**) or SKOV-3 CELLS (**C, D**) treatedd with KHYG-1 and TRAILv-KHTG-1 NK cells under various conditions. Quadrant gates define live cells (7AAD- Annexin V APC-), early apoptotic cells (7AAD- Annexin V APC+), late stage apoptotic cells (7AAD+ Annexin VAPC+) and necrotic cells (7AAD+ Annexin V APC-). **A**: OVCAR-3 cells after treated with non-modified KHYG-1 cells at 4 and 16 hours and E:T rations 0.1:1 and 10:1. **B**: OVCAR-3 cells after treated with modified TRAILv-KHYG-1 cells at 4 and 16 hours and E:T rations 0.1:1 and 10:1. **C**: SKOV-3 cells after treated with non-modified KHYG-1 cells at 4 and 16 hours and E:T rations 0.1:1 and 10:1. **D**: SKOV-3 cells after treated with modified TRAILv-KHYG-1 cells at 4 and 16 hours and E:T rations 0.1:1 and 10:1.


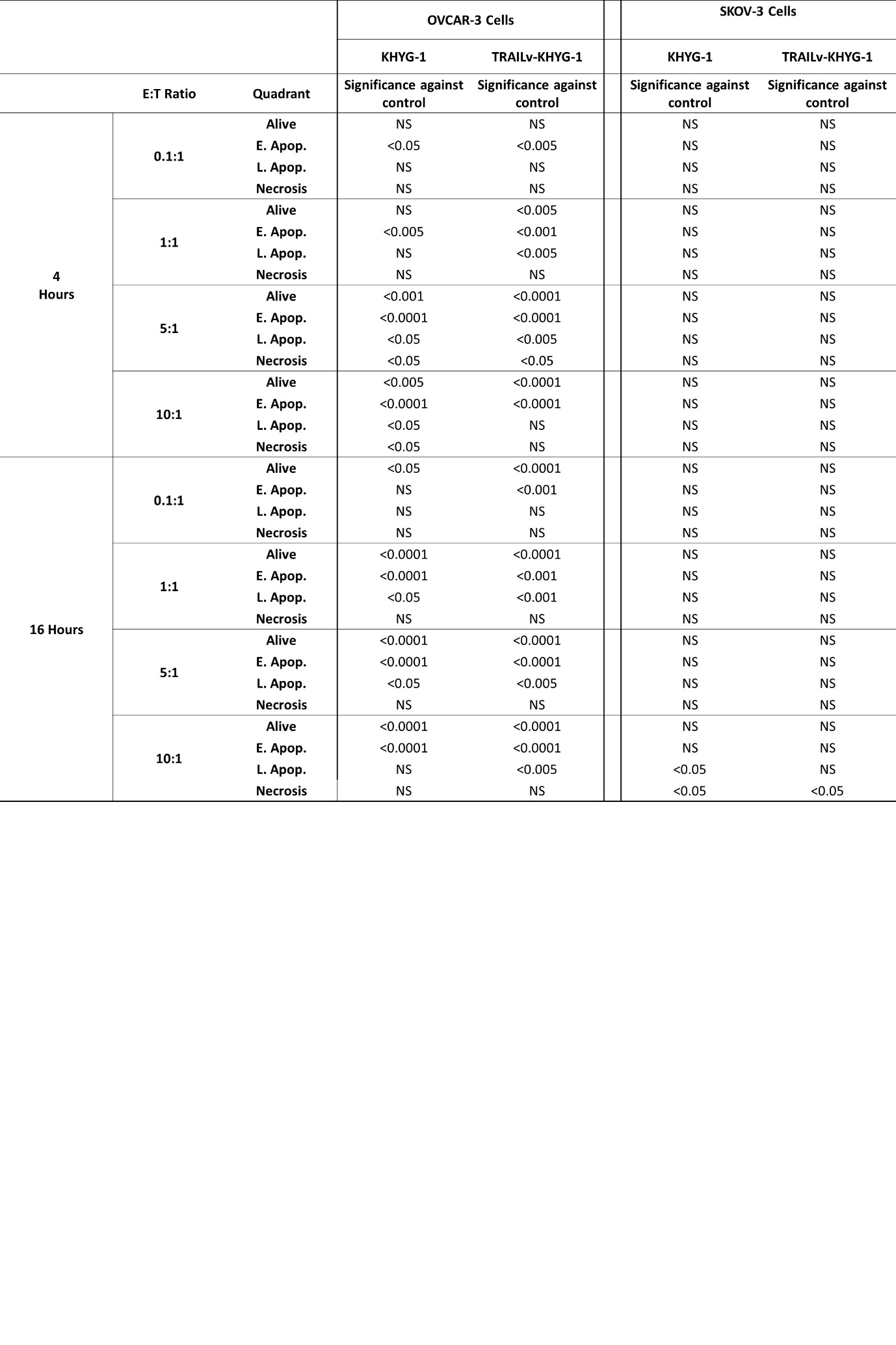


**Supplementary Table 1**: Statistical significance of OVCAR-3 and SKOV-3 treated with either KHYG-1 or TRAILv-KHYG-1 at 4 or 16 hours compared to non-treated control. E. Apop. = Early Apoptosis, L. Apop. = Late Apoptosis.
